# Identification of a novel platelet antagonist that binds to CLEC-2 and suppresses podoplanin-induced platelet aggregation and cancer metastasis

**DOI:** 10.18632/oncotarget.5811

**Published:** 2015-10-30

**Authors:** Yao-Wen Chang, Pei-Wen Hsieh, Yu-Tsui Chang, Meng-Hong Lu, Tur-Fu Huang, Kowit-Yu Chong, Hsiang-Ruei Liao, Ju-Chien Cheng, Ching-Ping Tseng

**Affiliations:** ^1^ Graduate Institute of Biomedical Sciences, College of Medicine, Chang Gung University, Taoyuan 333, Taiwan, Republic of China (ROC); ^2^ Graduate Institute of Natural Products, School of Traditional Chinese Medicine, College of Medicine, Chang Gung University, Taoyuan 333, Taiwan, Republic of China (ROC); ^3^ Department of Medical Biotechnology and Laboratory Science, College of Medicine, Chang Gung University, Taoyuan 333, Taiwan, Republic of China (ROC); ^4^ Graduate Institute of Pharmacology, National Taiwan University College of Medicine, Taipei 104, Taiwan, Republic of China (ROC); ^5^ Molecular Medicine Research Center, Chang Gung University, Taoyuan 333, Taiwan, Republic of China (ROC); ^6^ Department of Medical Laboratory Science and Biotechnology, China Medical University, Taichung 404, Taiwan, Republic of China (ROC); ^7^ Department of Laboratory Medicine, Chang Gung Memorial Hospital, Taoyuan 333, Taiwan, Republic of China (ROC)

**Keywords:** TCIPA, podoplanin, platelet aggregation, tumor metastasis

## Abstract

Podoplanin (PDPN) enhances tumor metastases by eliciting tumor cell-induced platelet aggregation (TCIPA) through activation of platelet C-type lectin-like receptor 2 (CLEC-2). A novel and non-cytotoxic 5-nitrobenzoate compound 2CP was synthesized that specifically inhibited the PDPN/CLEC-2 interaction and TCIPA with no effect on platelet aggregation stimulated by other platelet agonists. 2CP possessed anti-cancer metastatic activity *in vivo* and augmented the therapeutic efficacy of cisplatin in the experimental animal model without causing a bleeding risk. Analysis of the molecular action of 2CP further revealed that Akt1/PDK1 and PKCμ were two alternative CLEC-2 signaling pathways mediating PDPN-induced platelet activation. 2CP directly bound to CLEC-2 and, by competing with the same binding pocket of PDPN in CLEC-2, inhibited PDPN-mediated platelet activation. This study provides evidence that 2CP is the first defined platelet antagonist with CLEC-2 binding activity. The augmentation in the therapeutic efficacy of cisplatin by 2CP suggests that a combination of a chemotherapeutic agent and a drug with anti-TCIPA activity such as 2CP may prove clinically effective.

## INTRODUCTION

Metastasis is a highly complex process and the principle cause of cancer-associated death. Most cancer cells disseminated into the blood stream are rapidly eliminated by the high shear blood flow and by the host immune system. Less than 0.1% of cancer cells dislodged from the primary tumor site survives in the blood stream and causes metastasis [1]. The interaction between circulating tumor cells and platelets often results in tumor cell-induced platelet aggregation (TCIPA) that facilitates hematogenous tumor metastasis [1, 2]. A number of anti-TCIPA agents for blocking cancer metastases have been developed [3–6]. Aspirin, apyrase, tissue inhibitor of metalloproteinase-4, BM-567, XV454 and Abciximab are among the agents that inhibit tumor cell-platelet interactions and cancer metastases [7–11]. Treatment of cancer patients with these agents usually causes an increase in bleeding risk because most of the anti-TCIPA agents act on the platelet haemostatic proteins [4, 5, 12, 13]. Development of anti-TCIPA agents that do not interfere with physiological haemostasis is crucial for their utility as part of an anti-cancer therapeutic regimen.

Podoplanin (PDPN) is among the most frequently upregulated genes in squamous cell carcinoma, central nervous system tumors and germinal neoplasia [14, 15]. PDPN induces platelet aggregation by binding and activation of the C-type lectin-like receptor 2 (CLEC-2) [16, 17] leading to tyrosine phosphorylation of Src family kinases, Syk, and phospholipase C gamma 2 (PLCγ2) [18–22]. PDPN enhances metastatic foci formation and tumor progression without affecting tumor growth in animal studies [16]. The metastatic potency of cancer and the frequency of tumor cells embolized in the microvasculature of the lung are correlated with the platelet aggregation activity of PDPN [16, 23–25]. Specific inhibition of CLEC-2 signaling should not affect physiological haemostasis because CLEC-2-deficient platelets respond normally to the platelet agonists of collagen, ADP, U46619 and protease-activated receptor 4 peptide [26]. This indicates that PDPN-induced platelet-tumor cell interaction is a potential target for the development of an anti-metastases regimen.

This study involves an investigation of a novel small synthesized compound 2CP, a derivative of 4-*O*-benzoyl-3-methoxy-beta-nitrostyrene (BMNS), which specifically binds to CLEC-2 and inhibits PDPN-induced TCIPA. 2CP elicits and augments the therapeutic efficacy of anti-cancer drugs without affecting normal haemostasis in the mouse xenograft model. Novel signaling proteins transmitting PDPN-activated CLEC-2 signaling are also defined. The significance of these findings in the development of an anti-cancer regimen is discussed.

## RESULTS

### 2CP selectively inhibits PDPN-induced platelet aggregation

**Figure 1:**
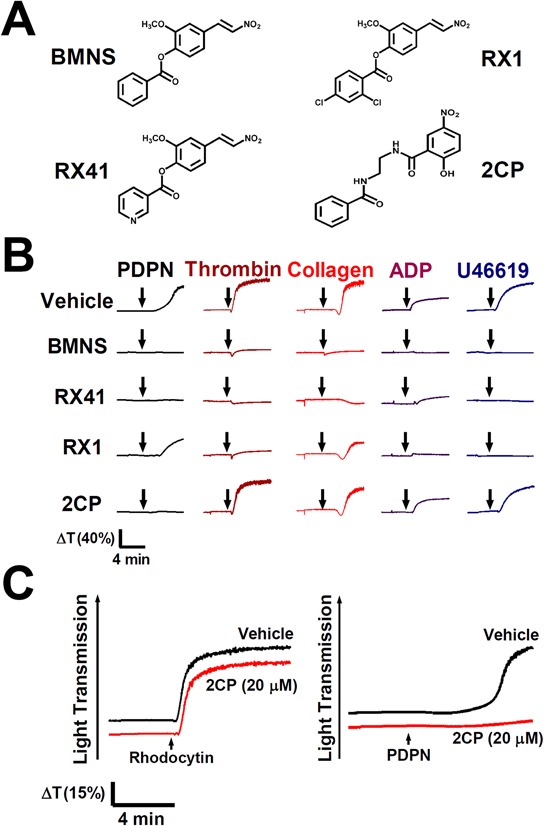
Selective inhibition of PDPN-induced platelet aggregation by 2CP **A.** Chemical structure of BMNS-related derivatives. **B.** Washed platelets were pre-incubated with the vehicle control DMSO or the indicated compounds (20 μM) at 37°C for 3 min and were subsequently stimulated by PDPN (8 μg/ml), thrombin (0.1 U/ml), collagen (2 μg/ml), ADP (20 μM) and U46619 (2 μM), respectively. Representative traces of platelet aggregation are shown. **C.** Washed platelets (1 × 10^9^/ml) were pre-incubated with the vehicle control DMSO or 2CP (20 μM) at 37°C for 3 min and were subsequently stimulated by the indicated CLEC-2 agonists (rhodocytin: 2 μg/ml; PDPN: 8 μg/ml). Platelet aggregation was recorded by a platelet aggregometer and representative traces of platelet aggregation for a total of at least four independent experiments are shown. Arrows indicate the point of agonist added.

BMNS, the non-selective inhibitor of agonist-induced platelet aggregation [27–31] was used as the lead compound to chemically synthesize BMNS-related derivatives RX1, RX41 and 2CP (Figure 1A, Supplementary method and Supplementary Figure S1). The effects of these compounds on agonist-induced platelet aggregation were analyzed. BMNS and RX41 non-selectively inhibited platelet aggregation induced by all agonists examined in this study including PDPN, thrombin, collagen, ADP and U46619 (Figure 1B). RX1 possessed moderate inhibition of platelet aggregation stimulated by PDPN or collagen, and strong inhibition on thrombin-, ADP- or U46619-stimulated platelet aggregation. 2CP inhibited PDPN-induced platelet aggregation with the IC50 equivalent of 12.1 ± 4.8 μM (Figure 1B and Table 1), but had little effect on platelet aggregation induced by the CLEC-2 agonist rhodocytin or either by thrombin, collagen, ADP or U46619 (Figure 1B and 1C). The IC50s of 2CP for these agonists were all >100 μM (Table 1). These results indicate that 2CP selectively inhibits PDPN-induced platelet aggregation.

**Table 1:** Effect of 2CP on platelet aggregation induced by various agonists

Compound	Conc. (μM)	Light transmission (% inhibition of control)^a^				
		PDPN	Thrombin	Collagen	ADP	U46619
Control	0	79.2 ± 1.9	78.3 ± 0.9	74.0 ± 1.2	34.6 ± 2.9	76.1 ± 1.7
2CP	100	8.4 ± 2.5** (89)	61.0 ± 4.3** (22)	60.0 ± 5.1* (18)	26.5 ± 2.9 (23)	65.6 ± 2.6** (13)
	20	19.8 ± 5.4** (75)	75.7 ± 0.9 (3.3)	69.3 ± 2.4 (6.3)	31.6 ± 2.2 (8.6)	72.6 ± 2.3 (4.5)
	5	59.8 ± 8.9 (24)	76.3 ± 1.0 (2.5)	70.7 ± 1.4 (4.4)	31.5 ± 3.2 (8.9)	74.3 ± 1.4 (2.3)
BMNS	20	5.8 ± 1.2** (92)	2.6 ± 2.6** (96)	0.1 ± 0.1** (99)	1.1 ± 2.4** (96)	0.5 ± 0.3** (99)

a The percentage of light transmission at the end of the aggregation assay are shown. The data represent the mean ± S.E (n ≧ 4).

* *p* < 0.05

** *p* < 0.01 when compared with the respective agonist control. The percentage inhibition of the light transmission when compared to the control is given in parentheses.

### 2CP inhibits PDPN-induced TCIPA

**Figure 2:**
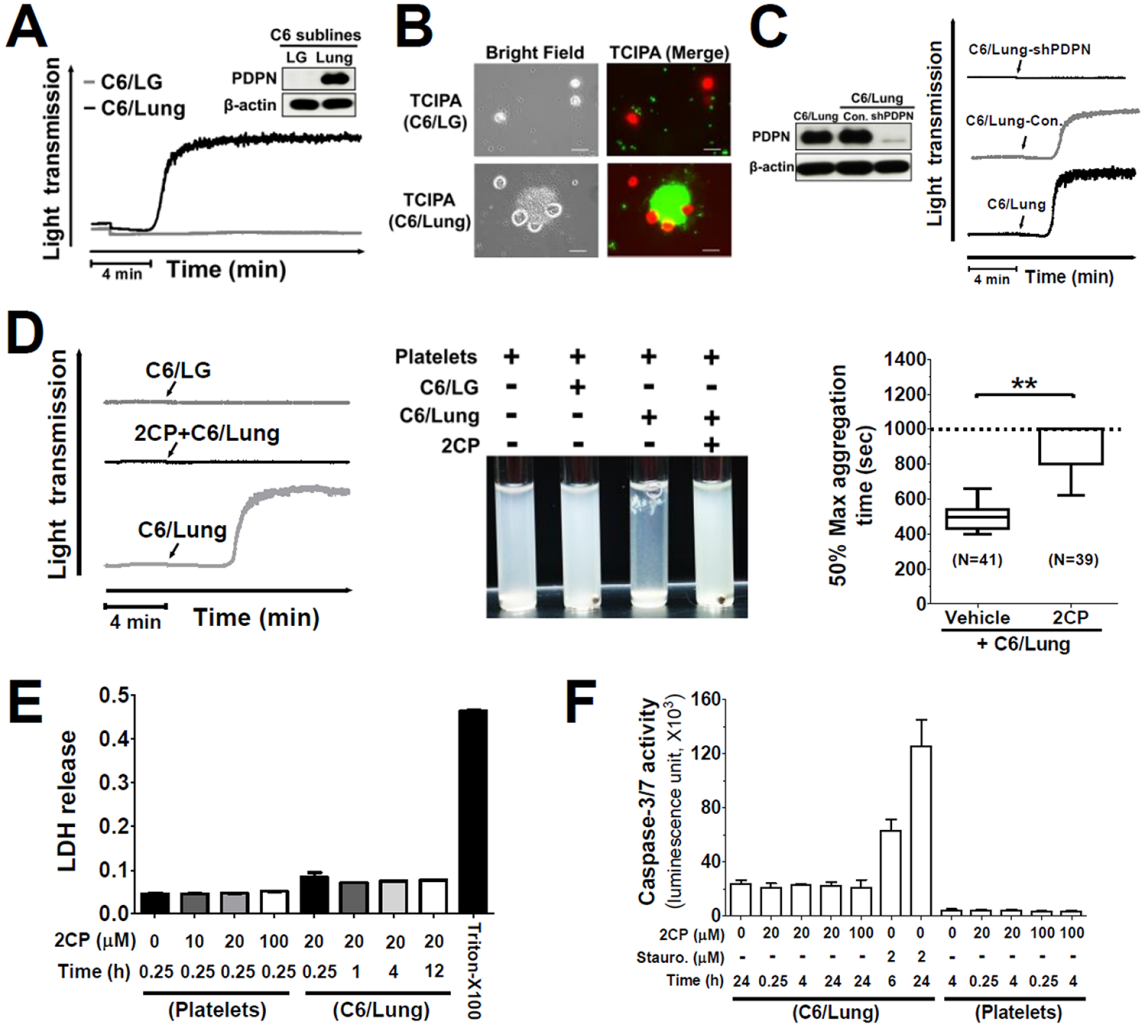
2CP inhibits PDPN-mediated TCIPA **A.** The expression of PDPN proteins in the indicated cell lines was determined by Western blotting using the anti-PDPN antibody (inserted panel). The expression of β-actin was used for the control of equal protein loading. The cells (1.5 × 10^6^) from the indicated cell lines were added to the human washed platelet suspension (1 × 10^9^/ml) to stimulate platelet aggregation. Tumor cell-induced platelet aggregation was measured and recorded by using an aggregometer. Representative traces of platelet aggregation are shown. **B.** Calcein-AM green-labeled platelets (1 × 10^9^/ml, green) were incubated with the calcein-AM orange/red-labeled cells (1.5 × 10^6^, red) in an aggregometer. The reaction mixtures were then placed on a glass slide for fluorescence microscopy analysis. Representative fluorescent images are shown to demonstrate the interaction between tumor cells and platelets. Scale bar = 20 μm. **C.** The expression of PDPN protein in the indicated cell lines was determined by Western blotting using the anti-PDPN antibody (left panel). The expression of β-actin was used for the control of equal protein loading. The platelet aggregation-inducing activities of these sublines were evaluated by TCIPA assays. Representative traces of platelet aggregation are shown (right panel). Arrows indicate the point of cells being added. **D.** The cells from the indicated cell lines were added into the washed platelets with or without pre-incubation with 2CP (20 μM). Representative traces of platelet aggregation (left panel) and the turbidity of the reactions (center panel) are shown. The time to reach 50% of the maximal aggregation was defined as the aggregation time that is shown as Box with whiskers (Min to Max) plot (right panel). The value is set to 1000 sec when no platelet aggregation was observed. **P < 0.01 when compared with the vehicle treatment. **E–F.** Platelets and C6/Lung cells were treated with the indicated concentrations of 2CP and the LDH and caspase 3/7 activities were measured. The data represent the mean ± S.E of three to six independent experiments.

C6/Lung cells were used as the cancer cell model to address whether 2CP is able to inhibit PDPN-induced TCIPA. This was based on the findings that C6/Lung cells expressed high levels of PDPN and induced platelet aggregation (Figure 2A) and the formation of tumor cell-platelet aggregates (Figure 2B). In contrast, the parental C6/LG cells did not express PDPN and were not able to induce platelet aggregation and form tumor cell-platelet aggregates (Figure 2A and 2B). Expression of PDPN short hairpin RNA in C6/Lung cells abrogated C6/Lung cells-induced platelet aggregation (Figure 2C), implying that PDPN is the key molecule mediating TCIPA.

**Figure 3:**
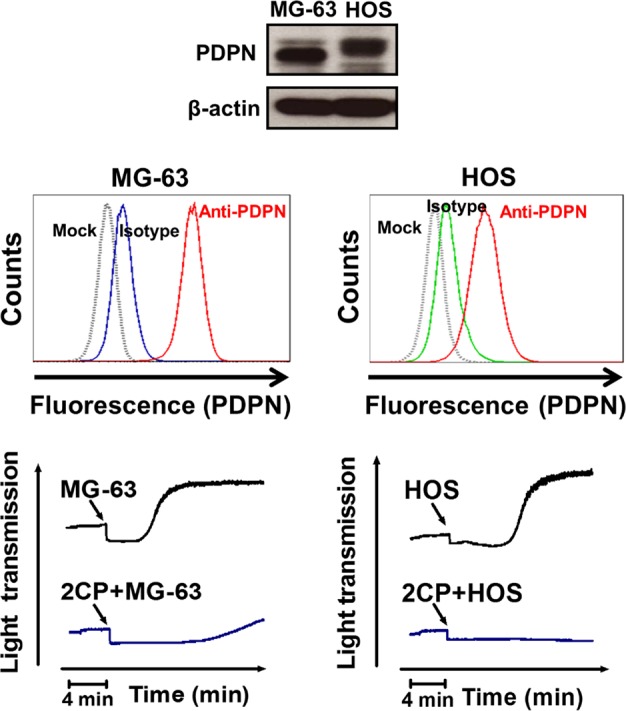
2CP inhibits platelet aggregation induced by MG-63 and HOS osteosarcoma cells PDPN expression in the MG-63 and HOS osteosarcoma cells was determined by Western blot (top panel) and flow cytometry (middle panel) analyses. For TCIPA assays, the MG-63 (2 × 10^6^) or HOS (1.5 × 10^6^) cells were added into the washed platelets (1 × 10^9^/ml) with or without pretreatment of 2CP (20 μM). Platelet aggregation was recorded by an aggregometer for 20 min (bottom panel).

TCIPA assays were performed by incubation of C6/Lung cells with human platelets in the presence or absence of 2CP. 2CP inhibited C6/Lung cells-induced platelet aggregation (Figure 2D, left panel) and caused the turbidity of the reaction mixture (Figure 2D, center panel). The time to reach 50% aggregation for the control and 2CP-treated group was 496.5 ± 65.4 sec (*n* = 41) and 910.6 ± 123.1 sec (*n* = 39), respectively (Figure 2D, right panel, *p* < 0.01). The lactose dehydrogenase (LDH) release and caspase 3/7 activity assays revealed that 2CP did not cause cytotoxic or apoptotic effects in platelets and tumor cells. This implies that cell stress or cell death does not account for the inhibitory activity of 2CP on TCIPA induced by C6/Lung cells (Figure 2E and Figure 2F). 2CP also inhibited TCIPA induced by the human osteosarcoma cell lines HOS and MG63, which express high levels of PDPN (Figure 3). These results indicate that 2CP inhibits PDPN-mediated TCIPA.

### Effects of 2CP on mouse tail bleeding time and pulmonary metastases in the mouse xenograft model

**Figure 4:**
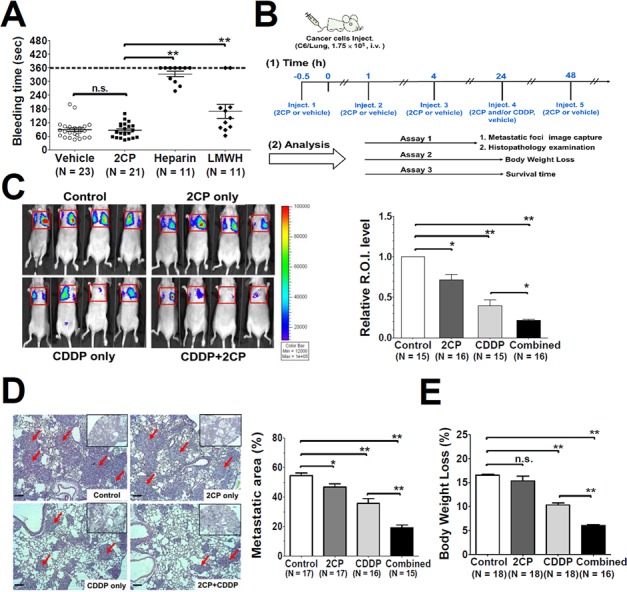
Effects of 2CP on tail bleeding time and mouse pulmonary metastasis **A.** The mice were intravenously injected with the control vehicle DMSO, 2CP (3.5 mg/kg), heparin (2 mg/kg) or LMWH (2 mg/kg) followed by measurement of the mouse tail bleeding time. The bleeding time was plotted and expressed as the mean ± S.E. A bleeding time longer than 360 sec was set as 360 sec. **B.** Timeline for the experimental protocols of the mouse pulmonary metastases model. The administration schedule and the therapeutic efficacy for 2CP and CDDP (2.5 mg/kg) were analyzed at the indicated time points. **C–E.** Representative bioluminescence images for tumor growth were shown (panel C, left) and quantified (panel C, right). Representative images of lung sections with metastatic foci (Hematoxylin and Eosin stain) were analyzed and quantified using Zeiss Axiovision software. Arrows point out the metastatic foci. (100 X magnification, scale bar = 100 μm). The corresponding metastatic area was expressed as the percentage of the whole lung region (panel D). The body weight was recorded and the percentage of body weight loss at day 21 after tumor inoculation was calculated. Data represent the mean ± S.E. from three to five independent experiments (panel E). *P < 0.05 and **P < 0.01 when compared with the control treatment. n.s., no significance.

The effects of 2CP on *in vivo* platelet function were evaluated by intravenous delivery of 2CP into the B6 mice followed by the tail bleeding time assay (Figure 4A). The dosage of 3.5 mg/kg that is equivalent to 5 times the Kd (24.5 ± 3.7 μM) for the binding of PDPN and CLEC-2 [18] was used in this assay. There was no difference in the bleeding time between the control (87.4 ± 8.1 sec, *n* = 23) and 2CP-treated (85.6 ± 7.1 sec, *n* = 21) mice (*p* = 0.87). The bleeding time of intravenous delivery of heparin and low-molecular-weight heparin (LMWH) was also compared with 2CP. At the dosage of 2 mg/kg (300 IU/kg for heparin and 200 IU/kg for LMWH), which was commonly used in the inhibition of cancer metastases in the mouse xenograft model [32–35], the tail bleeding time was 333.5 ± 11.7 sec (*n* = 11) and 169.9 ± 30.8 sec (*n* = 11) for heparin and LMWH, respectively. The bleeding time of the animals was significantly prolonged by heparin and LMWH when compared to the control and 2CP (*p* < 0.01). 2CP thereby elicits no deleterious effects on normal haemostasis.

The mouse xenograft model was then used to investigate whether 2CP has any effect on pulmonary metastases of C6/Lung cells. 2CP was injected into nude mice in the presence or absence of the anti-cancer agent cisplatin (CDDP) using the protocol shown in Figure 4B. Tumor formation was monitored by bioluminescence imaging analysis and histopathological examination of the lung tissue at day 15 after intravenous delivery of C6/Lung cells into the mice. Both 2CP (*p* < 0.05) and CDDP (*p* < 0.01) decreased the bioluminescence signal and the number of metastatic foci (Figure 4C and 4D). Combined treatment of the mice with 2CP and CDDP further decreased the bioluminescence signal and the number of tumor metastatic foci when compared with the mice treated with CDDP alone (*p* < 0.01). The metastatic area for the control, 2CP, CDDP, and the combined treatment of 2CP and CDDP was 54.4% ± 1.8% (*n* = 17), 46.7% ± 2.1% (*n* = 17), 35.6% ± 3.3% (*n* = 16), and 19.1% ± 1.8% (*n* = 15) of the surface area examined, respectively (Figure 4D).

**Figure 5:**
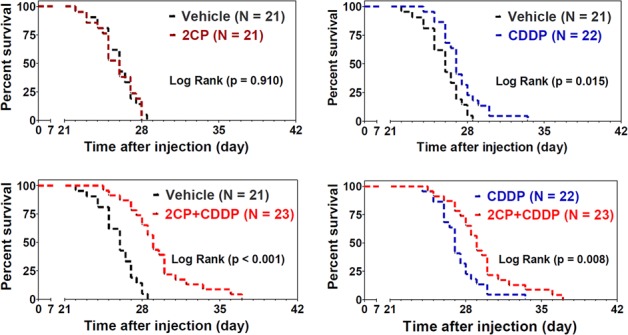
Combination of 2CP with CDDP prolongs the survival time of the experimental animals The mice were intravenously injected with the C6/Lung cells (1.75 × 10^5^) followed by the treatment with 2CP (*n* = 21), CDDP (2.5 mg/kg, *n* = 22), 2CP + CDDP (*n* = 23), or vehicle control (*n* = 21) as described in the Materials and Methods. The survival of the animals was recorded and the data were analyzed using the Kaplan-Meier survival curve and log rank test.

The body weight loss for each treatment group as recorded at day 21 after inoculation of cancer cells revealed that 2CP alone had no effect on the animals (Figure 4E). When combined with CDDP, 2CP reduced the degree of body weight loss concomitantly with the decrease in tumor burden (Figure 4E, *p* < 0.01). Consistent with these findings, 2CP alone did not extend the lifespan of the mice, while CDDP increased the survival of the animals (*p* = 0.015) when compared with the control group (Figure 5). Combined treatment of 2CP and CDDP caused a further increase in the lifespan of the mice when compared with the control (*p* < 0.001) or CDDP treatment alone (*p* = 0.008). In contrast to the *in vivo* effects of 2CP on the metastases of C6/Lung cells and animal survival, in C6/LG cells that did not express PDPN, 2CP did not prolong animal survival and did not decrease the lung metastatic foci formation (Supplementary Figure S2). These data imply that 2CP specifically inhibits the growth and metastases of cancer cells expressing PDPN.

We further address whether 2CP suppresses lung metastasis and increases animal survival when tumor injection preceded the delivery of 2CP into the mice (Supplementary Figure S3A). Our data revealed that combined treatment of mice with 2CP and CDDP slightly decreased the tumor burden (*p* = 0.07) but did not affect the percentage of body weight loss when compared to CDDP alone (Supplementary Figure S3B and S3C). 2CP was effective in prolonging animal survival. The survival fraction of the mice was 0%, 14.2%, 28.4% and 56.8% for the treatment with vehicle control, 2CP, CDDP, and the combined treatment of 2CP and CDDP, respectively (Supplementary Figure S3D). These data reinforce the notion that 2CP is effective in prolonging the survival of animals bearing PDPN-expressing tumors.

### 2CP reveals novel platelet CLEC-2 signaling

**Figure 6:**
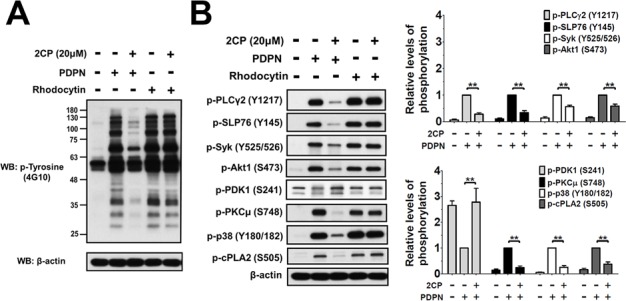
2CP selectively inhibits PDPN- but not rhodocytin-induced platelet signaling **A–B.** Washed platelets (1 × 10^9^/ml) were pre-incubated with or without 2CP (20 μM) at 37°C for 3 min, and then stimulated with PDPN (8 μg/ml) or rhodocytin (2 μg/ml). Whole cell lysates were prepared from the agonist-stimulated platelets and fractionated by SDS-PAGE. The protein phosphorylations were detected using the antibodies against phosphotyrosine (4G10) or the phospho-specific antibodies for the indicated proteins. The signals and relative phosphorylation levels of the indicated proteins were measured and quantified using the Image J software (NIH). The data represent the mean ± S.E. of four to eight independent experiments. **P < 0.01 when compared with the control treatment.

The effect of 2CP on PDPN- and rhodocytin-induced platelet protein tyrosine phosphorylation was analyzed and compared to determine the molecular basis of 2CP on the inhibition of PDPN-induced platelet aggregation and TCIPA (Figure 6A). Both PDPN and rhodocytin induced tyrosine phosphorylation of platelet proteins. Consistent with the differential effects of 2CP on PDPN- and rhodocytin-induced platelet aggregation (Figure 1C), only the phosphorylation induced by PDPN was suppressed by 2CP (Figure 6A). When the individual signaling proteins downstream of CLEC-2 were analyzed (Figure 6B), both PDPN and rhodocytin induced the phosphorylation of PLCγ2 (Y1217), SLP76 (Y145) and Syk (Y525/526). Other Syk-activated signaling proteins including Akt1 (S473), PKCμ (S748), p38 (Y180/Y182) and cytosolic phospholipase A2 (cPLA2-S505) were also phosphorylated after stimulation of platelets with PDPN and rhodocytin. 2CP inhibited PDPN- but not rhodocytin-induced phosphorylation of these proteins. On the other hand, pyruvate dehydrogenase kinase 1 (PDK1-S241) phosphorylation was decreased by PDPN and rhodocytin with 2CP reversing the effects of PDPN but not rhodocytin. These findings indicate that 2CP selectively suppresses PDPN-induced CLEC-2 signaling despite the fact that PDPN and rhodocytin share the same signaling proteins downstream of CLEC-2.

Whether 2CP directly targets the protein kinases that are downstream of CLEC-2 activation or are related to platelet activation was further analyzed by the *in vitro* protein kinase activity assays. 2CP had no effect on the activities of all 25 protein kinases analyzed (Supplementary Table S1). These data rule out the major protein kinases of platelet signaling as the direct targets of 2CP.

### Direct interaction of 2CP with CLEC-2

**Figure 7:**
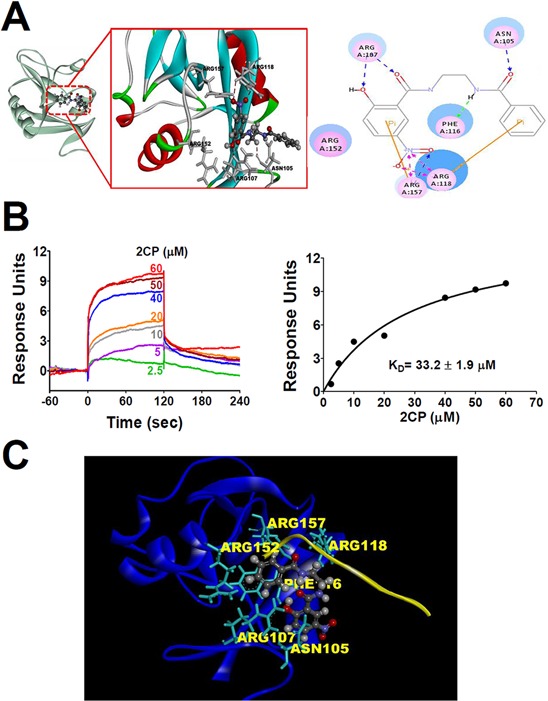
Molecular docking and SPR analyses reveals the interaction of 2CP with CLEC-2 **A.** Molecular docking modeling of 2CP with monomeric CLEC-2 was performed using the Discovery Studio program (Accelrys, San Diego, CA). 2CP is shown as a ball and stick model with gray carbons. Key residues of CLEC-2 are shown as sticks with gray carbons (left) or pink circles (right). Hydrogen bond interactions are shown as dotted red lines (left) or blue/pink lines. **B.** Different concentrations of 2CP were injected over the surfaces coupled with CLEC-2, producing a concentration-dependent signal. Sensorgrams from typical equilibrium-based binding experiments after subtraction of the background response from a control surface are shown in the left panel. Plot of the equilibrium binding response from the sensorgrams as a function of 2CP concentration is shown in the right panel. The curve is best fit to the experimental data with a calculated binding affinity of 33.2 ± 1.9 μM. Results are representative of four independent experiments. **C.** Podoplanin and 2CP share an overlapping binding site present in CLEC-2. The illustration was generated by superimposing the images of the crystal structure of CLEC-2 (blue)-PDPN (yellow) binding obtained from the PDB database (ID = 3WSR) and the molecular docking modeling of 2CP (ball and stick model with gray carbons) with monomeric CLEC-2. The key amino acids (Asn 105, Arg107, Phe116, Arg118, and Arg157) involved in the binding of 2CP are indicated (gray dashed lines represent hydrogen bonds). The model shows an overlying PDPN and 2CP-binding pocket in CLEC-2.

Computational molecular modeling was performed to elucidate whether 2CP directly binds CLEC-2. Docking analysis revealed that 2CP bound with the amino acid residues Asn105, Arg107, Phe 116, Arg118, and Arg157 of CLEC-2 by forming hydrogen bonds at side chain oxygen atoms (Figure 7A). The surface plasmon resonance (SPR) assay was performed to elucidate whether 2CP directly binds CLEC-2. A biosensor chip was coated with recombinant CLEC-2 and different concentrations of 2CP were flowed through the chip. Increasing concentrations of 2CP caused an increase in the binding to CLEC-2. The calculated binding affinity was equivalent of 33.2 ± 1.9 μM (Figure 7B). These results illustrated a direct interaction between 2CP and CLEC-2. Furthermore, we noted that the 2CP and PDPN binding sites on CLEC-2 overlapped when superimposing the crystal structures of PDPN and CLEC-2 with the docking model of 2CP and CLEC-2 (Figure 7C). The data further imply that 2CP inhibits CLEC-2 signaling by interfering with the binding of PDPN and CLEC-2.

## DISCUSSION

TCIPA plays a pivotal role in cancer metastases [1, 4, 5, 16, 36–38]. This study revealed that 2CP is the first synthetic compound that binds to CLEC-2 and inhibits PDPN-induced TCIPA without a deleterious effect on physiological haemostasis. 2CP in combination with an anticancer agent alleviates lung metastases in experimental animals and is a promising agent for anti-cancer metastases.

In addition to playing a role in TCIPA, PDPN is upregulated in the front end of the invasive margin of a tumor mass and is involved in the collective cell invasion process independent of the non-epithelial-mesenchymal transition of cancer cells [23, 39]. The expression of podoplanin in MDCK cells and oral squamous epithelial cancer cells increased single cell migration after loss of E-cadherin expression [40]. *In vitro* and *in vivo* studies using cell lines of different cancer origins support the notion that PDPN is crucial for cancer growth and metastases despite the fact of not knowing the molecular players and the mechanism that governs the decision by which of the two invasion patterns is activated by PDPN [16, 24, 41]. Clinical studies of human cancer tissues indicate that PDPN expression is associated with aggressive phenotypes of human cancer and is a poor prognostic marker in esophageal, oral, lung and cervical cancers [15, 39, 42–46]. PDPN is therefore a notable candidate for development of cancer-targeted therapy.

Various approaches for preventing PDPN/CLEC-2 interactions have been developed for anti-cancer therapy. Promising results using anti-PDPN antibody were demonstrated in several preclinical studies. The anti-PDPN antibodies NZ-1 and NZ-8 induced antibody-dependent cellular cytotoxicity and complement-dependent cytotoxicity in eliminating cancer cells and suppressing TCIPA, tumor growth and tumor metastases [47–49]. The chimeric anti-PDPN antibodies ChMS-1 and hP2–0 were developed to prevent cancer growth and metastases [25, 50]. The anti-PDPN antibodies NZ-1 that are either radiolabeled with N-succinimidyl 4-guanidinomethyl 3-[^131^I]iodobenzoate or fused to Pseudomonas exotoxin A carrying a C-terminal KDEL peptide exhibited significant suppression in the tumor growth of malignant glioma [51, 52]. The cost of antibody production and the potential immune response associated with antibody delivery limit the progress for clinical usage of these agents. 2CP has the potential for overcoming the costs associated with the usage of anti-PDPN antibodies because its simple chemical synthesis and its low molecular weight allows for its mass production. Based on the current study and the concept of rational drug design, a series of derivatives of 2CP can be synthesized to further improve the inhibitory efficacy of 2CP on PDPN-induced platelet aggregation associated with cancer growth and metastases.

2CP alone is not as effective as co-treatment of 2CP with CDDP in the experimental metastases model. This is explainable by the fact that host immunity or cytotoxic agents are required to eliminate cancer cells from the bloodstream to effectively inhibit metastases [5, 25, 52, 53]. 2CP is not cytotoxic and does not kill cancer cells. Instead, 2CP suppresses TCIPA and reduces the rate and the ratio of tumor embolization to the distant organs. Interfering with TCIPA is not sufficient to inhibit the growth of tumor cells and prevent the formation of metastatic foci. This notion is consistent with the observation that most anti-TCIPA agents are cytostatic, but not cytotoxic [4]. 2CP can therefore be considered as an adjuvant agent for cancer treatment in the presence of anti-cancer cytotoxic regimens.

The molecular basis of 2CP on the inhibition of PDPN-induced platelet aggregation and TCIPA is also addressed in this study. The parental compound of 2CP is a protein kinase inhibitor [30, 31]. Although the phosphorylation/activation of CLEC-2 signaling proteins such as Syk, PLCγ, and SLP76 are all suppressed in 2CP-treated platelets, 2CP does not appear to play a direct role as a protein kinase inhibitor. This is supported by the finding that 2CP has no effect on the *in vitro* activity of the protein kinases that are downstream of CLEC-2 signaling or are related to platelet activation. The differential effects of 2CP on platelet aggregation and protein phosphorylaton/activation induced by PDPN and rhodocytin, which share most of the CLEC-2 signaling pathways, further argue against the action of 2CP as a protein kinase or signaling protein inhibitor in platelets. An emerging question is: how does 2CP specifically inhibits PDPN- but not rhodocytin-induced platelet aggregation? Distinct binding properties of CLEC-2 with PDPN and rhodocytin were proposed despite the fact that the same amino acid residues Arg107, Arg118, Arg152, and Arg157 of CLEC-2 contribute to the interaction with both PDPN and rhodocytin [54]. Different sites of Arg118 in CLEC-2 are involved in the binding of the O-glycan of PDPN and the C-terminal Tyr136 of rhodocytin. 2CP interacts with the amino acid residues Asn105, Arg107, Phe116, Arg118 and Arg157 of CLEC-2. These results imply that 2CP interacts with the key amino acid residues (Arg 107, Arg 118 and Arg 157) involved in PDPN/CLEC-2 binding [54]. Hence, 2CP is likely to integrate into the same binding pocket or spatial location of PDPN and interfere with the binding of PDPN, but not rhodocytin, to CLEC-2. This binding model may explain the reason for the specific inhibition of PDPN-induced platelet aggregation and TCIPA by 2CP. Alternatively, the differential effect of 2CP on PDPN- and rhodocytin-induced CLEC-2 activation can be explained by the distinct binding affinity of CLEC-2 to PDPN and CLEC-2 to rhodocytin, which is equivalent to 24.5 μM and 1 μM, respectively [18, 55]. 2CP is likely to compete more efficiently in the interaction of CLEC-2 with PDPN than in the interaction with rhodocytin. Both models explain the specific inhibition of PDPN-induced platelet aggregation and TCIPA by 2CP.

In addition to the activation of the known signaling proteins Syk, SLP76 and PLCγ2, PDPN and rhodocytin induce the phosphorylation of Akt1 (S473), PKCμ (S748), p38 (Y180/Y182), and cPLA_2_ (S505) but suppress the phosphorylation of PDK1 (S241). 2CP selectively reverses the phosphorylation status of these protein kinases induced by PDPN in association with the selective inhibition of PDPN-induced platelet aggregation. These findings are consistent with previous studies of various signaling pathways suggesting that Akt, PKCμ, p38, and cPLA_2_ are the Syk downstream effectors [56–59]. p38 phosphorylation was observed in rhodocytin-triggered CLEC-2 activation of monocytes/macrophages [60]. BCR-mediated p38 activation is Lyn- and Syk-dependent [57]. Syk also regulates FcγRIIA-induced LAT phosphorylation and activation of PI3K/Akt and p38 [61]. PKCμ coprecipitates with both Syk and PLC-γ1/2 [59], and is activated in the DT40 B cell line in a Syk-dependent manner [57]. cPLA_2_ activation by β-glucan is dependent on the activation of Syk in macrophages [62]. PDK1 (S241) phosphorylation is suppressed during PDPN-induced platelet aggregation which can be reversed by 2CP. Autophosphorylation at Ser241 is important for PDK1 kinase activity, which is negatively regulated by binding to 14–3-3 through the PDK1 autophosphorylation site Ser241 [63]. While PI3K/PDK1/Akt signaling is the downstream effectors of Syk [58, 61, 64] and is related to platelet activation and aggregation [65, 66], the phosphorylation at Ser241 and the binding to 14–3-3 are most likely involved in the control of PDPN-induced PDK1 activity. These findings lay the foundation for uncovering novel signaling proteins upon CLEC-2 activation.

In conclusion, a new and non-cytotoxic small molecule compound 2CP was synthesized that binds to CLEC-2 and inhibits PDPN-induced CLEC-2 activation, platelet aggregation and TCIPA. Novel therapeutic usage of 2CP and the identification of notable CLEC-2 signaling molecules were found by analyzing the effect of 2CP on metastases in experimental animals and on PDPN-induced platelet activation. 2CP has potential as an anti-metastatic agent in cancer and as a tool for elucidating the molecular mechanism of PDPN-induced CLEC-2 activation.

## MATERIALS AND METHODS

### Reagents and antibodies

Thrombin and ADP were purchased from Calbiochem (Darmstadt, Germany). Collagen was purchased from Chrono-Log Co. (Havertown, PA). The recombinant human PDPN was purchased from Sino Biological Inc. (Beijing, China). Rhodocytin (aggretin) was purified from *Calloselasma rhodostoma* venom as described previously [60]. U46619 was purchased from Cayman Chemical (Ann Arbor, MI). Apyrase, prostacyclin (PGI_2_), and bovine serum albumin (BSA) were purchased from Sigma (St Louis, MO). Fluorescence cell trace calcein dyes (green calcein-AM and red/orange calcein-AM) were purchased from Invitrogen (Carlsbad, CA). The Alexa Fluor 488-conjugated anti-human PDPN antibody was purchased from BioLegend Inc. (San Diego, CA). Heparin (unfractionated) was kindly provided by Dr. Hsiang-Ruei Liao (Chang Gung University, Taiwan). LMWH (Enoxaparin, Clexane) was obtained from Sanofi Pharma Inc. (Maisons-Alfort Cedex, France). CDDP was kindly provided by Dr. Jo-Chi Tseng (Chang Gung Memory Hospital, Taiwan). The anti-phosphotyrosine monoclonal antibody (4G10) was purchased from EMD Millpore (Billerica, MA). Anti-phospho-specific antibodies for PLCγ2 (Y1217), SLP-76 (Y145), Syk (Y525/526), Akt1 (S473), PDK1 (S241), PKCμ (S748), cPLA_2_ (S505) and p38 (Y180/182) were purchased from Cell Signaling Technology Inc. (Beverly, MA). The anti-rat PDPN monoclonal antibody and anti-β-actin antibody was purchased from Novus Biological (Mill Valley, CA). The LDH activity assay kit was purchased from Biochain Inc. (Hayward, CA). The caspase activity assay kit (Caspase-Glo 3/7 kit) was purchased from Promega Inc. (Madison, WI)

### Animals, cell lines and compound supply

All animals and experimental protocols were approved by the Institutional Animal Care and Use Committee (IACUC) at Chang Gung University (Taiwan, Republic of China) with the approval ID CGU10–004 and CGU12–057. BALB/c nude mice were purchased from National Laboratory Animal Center (Taipei, Taiwan) and housed under pathogen-free conditions. Rat glioma cell line (C6, BCRC-860046) and human osteosarcoma cell lines (MG63, BCRC-60279; HOS, BCRC-60308) were purchased from Bioresource Collection and Research Center (BCRC, Taiwan). C6/LG was derived from the rat C6 glioblastoma cells by expressing fusion reporter genes of luciferase and green fluorescent protein (LG). C6/Lung is a subline of C6/LG obtained by recovery of the lung metastatic colonies that were formed when C6/LG cells were intravenously injected into the nude mice. All small molecule compounds under study were synthesized as described in the supplementary methods.

### Human blood sampling

All experimental protocols and procedures were approved by the Institutional Review Board of Chang Gung Memorial Hospital (Linkou, Taiwan, Republic of China) with the approval ID 99–0404B and 101–3497B. Healthy volunteers without history of hematological diseases, platelet or coagulation disorders, or taking medication that might influence hematological function were recruited for this study and signed the written informed consent.

### Preparation of washed human platelets

Fresh blood was drawn from informed healthy volunteers without taking any drugs at least two weeks prior to this study. For preparation of washed human platelets [67], platelet-rich plasma (PRP) was obtained from whole blood mixed with 3.2% trisodium citrate (9:1) by centrifugation at 200 g for 20 min. The platelets were then prepared from PRP by centrifugation (900 g, 10 min) and washed twice with Tyrode's buffer (137 mM NaCl, 2.65 mM KCl, 12 mM NaHCO_3_, 0.43 mM NaH_2_PO_4_, 2 mM CaCl_2_, 1 mM MgCl_2_, 5 mM glucose, 5 mM HEPES, pH 7.35) containing 0.5 μM PGI_2_ and 0.2 unit/ml apyrase. Finally, washed platelets were re-suspended in Tyrode's buffer and adjusted to 3 × 10^8^/ml for platelet aggregation or 1 × 10^9^/ml for PDPN- or tumor cells-induced platelet aggregation assays.

### Platelet aggregation and TCIPA

Washed platelet suspension was pre-incubated with the solvent vehicle or test compounds for 3 min at 37°C with stirring at 900 rpm [67]. Then, platelet aggregation was triggered by the addition of PDPN (4 μg), thrombin (0.1 U/ml), collagen (2 μg/ml), U46619 (2 μM), ADP (20 μM) or tumor cells (1.5 × 10^6^ cells). The platelet aggregations and TCIPA were monitored and recorded for 15–20 min by using an aggregometer with Aggro/Link processing software (Chrono-Log Corp., Havertown, PA). The time to reach 50% maximal aggregation was recorded and defined as the aggregation time.

For observation of tumor cell-platelet aggregation by fluorescent microscopy, platelets or tumor cells were pre-loaded with 2 μM fluorescence cell trace dyes (green calcein-AM for platelets and red/orange calcein-AM for tumor cells) at 37°C for 30 min, then washed, re-suspended, and adjusted the cell number for use. Briefly, calcein-AM green-labeled platelets (1 × 10^9^/ml, green) were incubated with the calcein-AM orange/red-labeled cells (1.5 × 10^6^, red) in a reaction tube to trigger TCIPA in an aggregometer. The reaction was terminated when the degree of aggregation for C6/Lung cells reached 30% of the light transmission. All reaction mixtures were removed from the tube and placed on a glass slide for fluorescence analysis. The fluorescence hetero-aggregates of platelets and tumor cells were examined by using a Zeiss Axiovert 200M microscope (Carl Zeiss, Germany).

### Cytotoxicity assays with lactose dehydrogenase release and caspase activity measurement

The LDH activity assay kit was used to evaluate the cytotoxic effects of 2CP on platelets and culture cells. The assay was performed as described by the manufacturer. Briefly, platelets (1 × 10^9^/ml) were incubated with 2CP (10–100 μM) and stirred at 900 rpm for 15 min. The supernatants for LDH measurement were collected by first centrifugation at 3000 g for 5 min followed by another centrifugation at 10,000 g for 5 min. For tumor cells (1 × 10^6^), monolayer confluent cells were treated with 2CP (20 μM) for 0–12 h and the supernatants were collected by centrifugation as described above. Supernatants (100 μl) were mixed in an optical 96-well flat microplate with 45 μl of the assay reaction mixture containing diaphorase, NAD^+^, tetrazolium INT and lactate. After incubation at room temperature for 30 min, the reaction was stopped by adding 50 μl of stop solution. The LDH activity of the sample was determined by measuring the absorbance at the wavelength of 490 nm. As a control, the supernatant from the platelets/cells that were lysed by 1% triton X-100 was used as a control for measuring the total amount of LDH.

The measurement of caspase activity was performed using a Caspase-Glo 3/7 assay kit according to the manufacturer's instructions. Briefly, platelets (1 × 10^8^) or tumor cells (4 × 10^3^) were placed in a 96-well white microplate in the absence or presence of 2CP (20 or 100 μM) for various time points (0, 0.25, 4 and 24 h) at 37°C. Then an equal volume of Caspase-Glo assay reagent was added to the cells. The reaction mixtures were incubated for 1 h at 37°C. The luminescence signal was measured by using a SpectraMax luminometer (Molecular Devices). Cells treated with staurosporine (2 μM) were used as a positive control for caspase activity.

### Experimental pulmonary metastases, body weight loss and animal survival analyses

Cancer cells (1.75 × 10^5^/100 μl) were intravenously injected into 7–8 week old BALB/c-nu/nu mice. The test compounds were intravenously injected into the mice (2CP, 3.5 mg/kg; and CDDP, 2.5 mg/kg). At day 15 post-injection of tumor cells, *in vivo* bioluminescence imaging analysis of lung metastatic foci were measured using a Xenogen IVIS-100 fluorescence image system (Xenogen, Alameda, CA). The relative region of intensity (R.O.I) was analyzed for tumor growth. Lung tissue was extirpated and stained with hematoxylin and eosin for histologic examination by a Zeiss PrimoStar microscope (Carl Zeiss, Gottingen, Germany). The metastatic areas of the lung were analyzed and quantified using Zeiss AxioVision software (Carl Zeiss, Germany). The corresponding metastatic area was expressed as a percentage of the whole lung. The body weight loss was recorded at day 21 after tumor inoculation. The percentage of the weight difference was calculated using the equation of [(Day 0 - Day 21)/Day 0] x 100%. The survival of the animals was recorded and the data were analyzed using the Kaplan-Meier survival curve and log rank test.

### Tail bleeding time assay

The tail bleeding time assay was performed as described previously [67–68]. Briefly, mice (7–8 weeks old) were intravenously injected with 2CP (3.5 mg/kg), heparin (2 mg/kg, equivalent to 300 IU/kg), LMWH (2 mg/kg, equivalent to 200 IU/kg) or vehicle control. Ten min after injection, mice were anesthetized and 0.5 cm of the distal tail was cut with a scalpel. The amputated tail was immediately immersed and placed in physiological saline pre-warmed to 37°C. The bleeding time was recorded from the moment of transection until blood flow ceased. Observations were stopped at 360 sec even if bleeding did not cease. A bleeding time longer than 360 sec was set as 360 sec.

### Western blot analysis

For isolation of platelet lysates, the platelets were mixed with 5X ice-cold lysis buffer (100 mM Tris-HCl, pH 7.5, 300 mM NaCl, 5% Triton X-100, 5 mM EGTA, 5 mM EDTA, 5 mM PMSF, 5 mM Na_3_VO_4_, 20 μg/ml leupeptin, and 20 μg/ml aprotinin) and kept on ice for 4 h [67]. Proteins were solubilized by adding sample buffer to the final concentration of 60 mM Tris-HCl pH 6.8, 2% SDS, 10% glycerol, 0.1% bromophenol blue, and 50 mM DTT. The lysates were then boiled at 95°C for 10 min followed by sodium dodecyl sulfate-polyacrylamide gel electrophoresis (SDS-PAGE). For isolation of cell lysates, cultured cells (2 × 10^6^/ml) were harvested and washed twice with ice-cold 1X PBS. Then, cells were solubilized in 1X ice-cold lysis buffer [69] and kept on ice for 4 h. After first centrifugation at 3,000 g for 10 min to remove cell debris, the supernatants were collected for second centrifugation (10,000 g, 5 min) followed by protein quantification.

The extracted proteins were fractionated by SDS-PAGE and transferred to PVDF membrane (Pall Corp., Ann Arbor, MI) using the Bio-Rad electrotransfer system. The membranes were blocked with 5% non-fat dry milk at 4°C overnight and incubated with the indicated primary antibody that recognizes the desired targets. After washing with Tris-buffered saline containing 0.1% Tween 20 (TBS-T) four times, the membranes were incubated with the HRP-conjugated secondary antibody (1:20000) for 45 min at room temperature. After washing twice with TBS-T, the proteins were detected by using an enhanced chemiluminescence detection kit (Millipore Corp., Bedford, MA).

### Candidate compound molecular docking modeling

For docking studies, the three-dimensional structure of 2CP was constructed using a Chem 3D ultra 9.0 software Chemical Structure Drawing Standard (CambridgeSoft Corporation, Waltham, MA). The crystal structure of CLEC-2 (PDB ID 3WSR) was retrieved from the RCSB Protein Data Bank. The water molecules were discarded, and the hydrogen atoms were added to the protein with Discovery Studio 4.0 (Accelrys, San Diego, CA). The forcefield CHARMm function was used to roughly search the conformations when compounds docked on to CLEC-2, and then the modified active site of CLEC-2 was selected as the binding site for the study. The conformations of 2CP were optimized using the same force field function, and then flexibly docked in a stepwise manner with the protocol of Dock ligands (Libdock) in Discovery Studio 4.0 and LibDock scores as scoring functions.

### Surface plasmon resonance binding assay

The binding affinity of 2CP to CLEC-2 was measured with a Biacore SPR instrument Biacore T200 (GE Healthcare, Uppsala, Sweden). The amount of bound compound with the CLEC-2 protein was reported as biosensor response units (RU). All assays were performed at 25°C in PBS-P/1% DMSO running buffer (0.005% P20 surfactant and 1% DMSO in PBS buffer). Non-specific binding was eliminated and controlled by subtraction of the reference flow cell signal and running buffer wash. K_D_ values and sensorgrams were analyzed with Biacore Evaluation software 1.0 (GE Healthcare). For kinetic binding experiments, recombinant CLEC-2 (20 μg/ml) was immobilized on a CM5 biosensor chip surface by amine-coupling (10 mM acetate, pH 5.0) according to the manufacturer's protocol. Then, various concentrations (2.5 to 60 μM) of 2CP were injected for 2 min at 30 μl/ml flow rate onto the pre-immobilized CLEC-2 chip surface to record the 2CP-CLEC-2 interaction followed by calculating the equilibrium dissociation constant (K_D_).

### Statistical analysis

All experiments were repeated at least three times and results were expressed as means ± S.E. Differences between the sample-treated group and control group were analyzed by unpaired Student's *t*-test, Mann-Whitney *U*-test, and one-way ANOVA using the Prism statistical software version 4.0 (San Diego, CA, USA), where appropriate. The survival rate of the animals was examined by the Kaplan-Meier curve and log rank test. Results were considered statistically significant at *a* value of *P* < 0.05.

## SUPPLEMENTARY METHODS FIGURES AND TABLE



## Abbreviations

BMNS: 4-O-benzoyl-3-methoxy-beta-nitrostyrene
CDDP: cisplatin
CLEC-2: C-type lectin-like receptor 2
cPLA2: cytosolic phospholipase A2
LDH: lactose dehydrogenase
LMWH: low-molecular-weight heparin
PDK1: pyruvate dehydrogenase kinase 1
PDPN: podoplanin
PGI2: prostacyclin I2
PKCμ: protein kinase C mu
PLCγ2: phospholipase C gamma 2
PRP: platelet-rich plasma
SDS-PAGE: sodium dodecyl sulfate-polyacrylamide gel electrophoresis
SPR: surface plasmon resonance
TCIPA: tumor cell-induced platelet aggregation.
