# Supplementary material for: Identification of a novel platelet antagonist that binds to CLEC-2 and suppresses podoplanin-induced platelet aggregation and cancer metastasis
Source: Oncotarget. 2015 Oct 30;6(40):42733–48. doi: 10.18632/oncotarget.5811 (PMC4767466; doi:10.18632/oncotarget.5811)
Supplement: Supplementary file 1 [file oncotarget-06-42733-s001.pdf]

SUPPLEMENTARY METHODS

Synthesis and characterization of 2CP

2CP was synthesized as outlined in the Supplementary Figure S1. Chloro-4-nitro-2-(trifluoromethyl) benzene (13.3 mmole) was dissolved in dimethyl sulfoxide (12 mL), and then sodium hydroxide (40 mmole) was slowly added at a temperature lower than 25 ºC. The reaction mixture was stirred at room temperature for 8 h, and then 1 N HCl solution was added to adjust the pH values to 1~2. The solution was partitioned between dichloromethane and water, and the organic layer was evaporated at reduced pressure to yield residues. The residues were subjected to purification on a silica gel column (50 g) and eluted with $CHCl_3$/*n*-hexane (2:1) to yield 4-nitro-2-(trifluoromethyl) phenol (1.85 g). 4-Nitro-2-(trifluoromethyl) phenol (500 mg, 2.4 mmole) and *tert*-butyl 2-aminoethylcarbamate (769 mg, 4.8 mmole) which were dissolved in 1 M aqueous NaOH solution (7.2 mmole) and dioxane (10 mL) and stirred at 100 ºC for 24 h. 1 N HCl solution was added to adjust the pH value to 1~2, and then extracted with CH_2Cl_2. five times (each for 20 ml). The obtained CH_2Cl_2 solution was hydrated over $MgSO_4$ and concentrated under vacuum. The organic layer was evaporated at reduced pressure, and subjected to a silica gel column (50 g) and eluted with the system of $CHCl_3$/*n*-hexane (19:1) to afford *tert*-butyl 2-(2-hydroxy-5-nitrobenzamido) ethylcarbamate (600 mg). *tert*-Butyl 2-(2-hydroxy-5-nitrobenzamido) ethylcarbamate was deprotected in 20% trifluroacetic acid (TFA) solution (TFA:DCM = 1:4) at room temperature for 2 h, and was purified on a silica gel column and eluted using the system of $CHCl_3$/*n*-hexane (4:1) to give *N*-(2-aminoethyl)-2-hydroxy-5-nitrobenzamide (390 mg). *N*-(2-aminoethyl)-2-hydroxy-5-nitrobenzamide (390 mg) was dissolved in 2 N NaOH (10 ml) and reacted with benzoyl chloride at room temperature for 16 h. The mixture was then concentrated under vacuum. The residue was subjected to purification on a silica gel column and eluted using the system of $CHCl_3$/*n*-hexane (30:1) to afford 2CP (325 mg). ^{1}H NMR (400 MHz, acetone-*d6*): δ9.12 (1H, *s*, NH), 8.76 (1H, *d*, *J* = 2.4 Hz, H-2), 8.29 (1H, *dd*, *J* = 2.4, 9.2 Hz, H-4), 8.17 (1H, *s*, NH), 7.90 (2H, *d*, *J* = 7.2 Hz, H-8, 12), 7.51 (1H, *t*, *J* = 7.2 Hz, H-10), 7.44 (2H, *t*, *J* = 7.2 Hz, H-9, 11), 8.29 (1H, *d*, *J* = 9.2 Hz, H-5), 3.70 (4H, *t*, *J* = 2.8 Hz, H-14, 15). ^{13}C NMR (acetone-*d6*, 100 MHz) δ: 170.4 (*s*, C=O, C-16), 168.6 (*s*, C=O, C-13), 168.3 (*s*, C-6), 140.7 (*s*, C-3), 135.9 (*s*, C-7), 132.4 (*s*, C-10), 130.1 (*s*, C-4), 129.5 (*s*, C-9, 11), 128.4 (*s*, C-8, 12), 124.7 (*s*, C-2), 119.9 (*s*, C-5), 115.4 (*s*, C-1), 41.5, 40.2 (*s*, C-14, 15). ESI-MS *m*/*z* 330 (100) $[M+H]^+$, 352 (32) $[M+Na]^+$. HRESI-MS *m*/*z* 352.0911 (calc.: 352.0909; $C_{16}H_{15}N_3O_5Na$).

SUPPLEMENTARY FIGURES AND TABLE

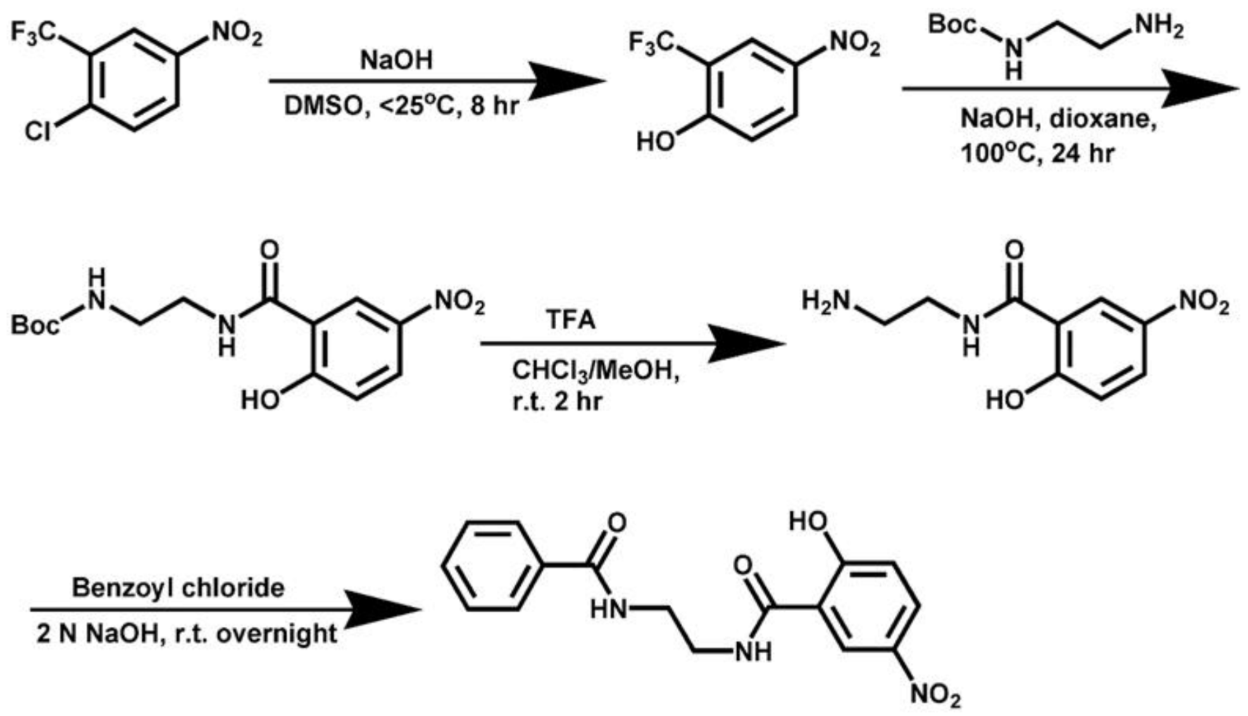

Supplementary Figure S1: The chemical reaction for the synthesis of 2CP. The detailed procedures for synthesis of 2CP were described in the supplementary methods.

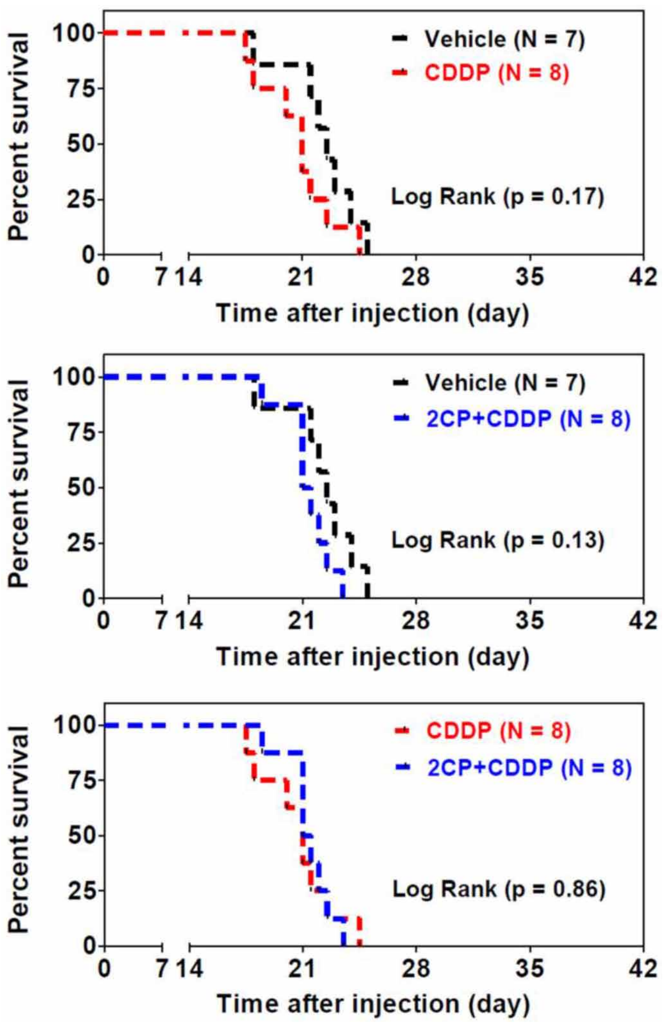

Supplementary Figure S2: 2CP does not prolong the survival of animals injected with PDPN-deficient C6/LG cells. The nude mice were intravenously injected with the C6/LG cells (1.5×10^5) followed by the treatment of CDDP ($n = 8$), 2CP + CDDP ($n = 8$), or vehicle control ($n = 7$) as described in the Materials and Methods. The survival time of the animals was recorded and analyzed using the Kaplan-Meier survival curve and the log rank test.

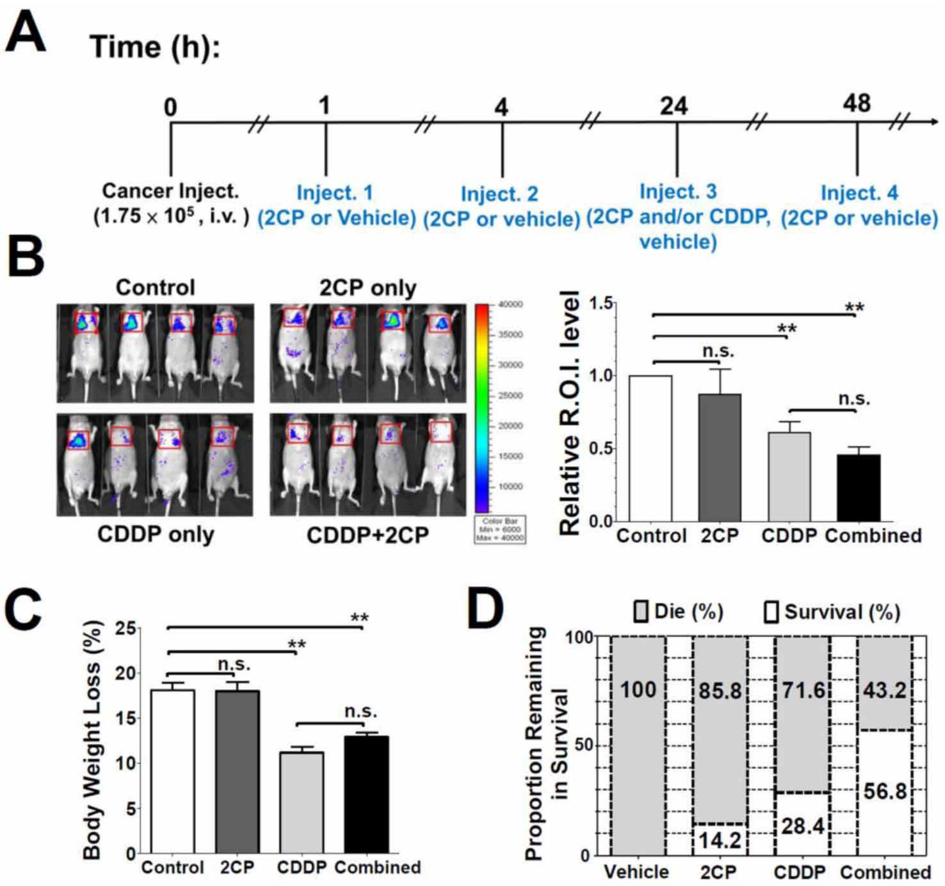

Supplementary Figure S3: The effect of post-treatment of 2CP on mouse pulmonary metastases and animal survival. **A.** Timeline for the experimental protocols of the mouse pulmonary metastases model. The post-administration schedule of 2CP and the therapeutic efficacy were analyzed at the indicated time points as described in the "Materials and Methods". **B.** The mice intravenously injected with C6/Lung cells (1.75×10^5 cells) were subject to the indicated treatment of vehicle control, 2CP (3.5 mg/kg), CDDP (2.5 mg/kg), and 2CP + CDDP, respectively. Representative bioluminescence images for tumor formation were shown (left panel) and quantified (right panel). **C.** The body weight recorded at day 21 after tumor inoculation was used to calculate the percentage of the body weight loss. Data represent the mean ± S.E. of seven mice. $^{**}P < 0.01$ when compared with the control treatment. n.s., no significance. **D.** The data show the percentage of animal survival at Day 40 after the initiation of experiments.

Supplementary Table S1: Effect of 2CP on selected protein kinase activity

Kinases	Kinase activity (% control)[a]
CaMKI	92
CaMKIIβ	98
cSrc	98
FAK	86
Fyn	109
Hck	97
Lyn	93
PDK1	104
PKBα	103
PKBβ	110
PKCδ	100
Syk	95
Yes	91
ZAP-70	90
PI3 Kinase (p110α)	100
PI3 Kinase (p110β)	99
PI3 Kinase (p120γ)	92

[a] The effect of 2CP (50 μM) on the selected protein kinase activities was analyzed using the Millipore Kinase Profiler service (Radiometric filter binding assay). The data represent the mean of replicate assays.
